# A Systematic Analysis of the Nasal Septum in Crooked Noses and Suggested Treatment Algorithm According to Preservation Rhinoplasty (PR) Principles

**DOI:** 10.1007/s00266-023-03293-3

**Published:** 2023-02-24

**Authors:** Mathias Tremp, Jonas Schneider, Ram Badari Narayan Raghu, Abdulkadir Goksel, Yves Saban

**Affiliations:** 1 Faculty of Medicine, University of Basel, Basel, BS Switzerland; 2 Private Practice, Hirslanden Private Hospital Group, Dorfplatz 1, 6330 Cham, Switzerland; 3 Gurugram-Narayana Superspeciality Hospital, Gurugram, India; 4 RinoIstanbul Facial Plastic Surgery Clinic, Istanbul, Turkey; 5 Rhinoplasty Private Practice, Nice, France

**Keywords:** Rhinoplasty, Septum, Deviation, Crooked noses, Septoplasty

## Abstract

**Background:**

A deviated nose is a common problem among patients for both cosmetic and functional reasons. The correction remains a major challenge for the rhinoplasty surgeon. Unrecognized nasal septal deviations stand as the primary reason for failed rhinoplasty outcomes. There is a paucity of data in the literature about septoplasty classifications and technical details in preservation rhinoplasty (PR) for various crooked noses.

**Materials and Methods:**

The aim of this article is to provide a comprehensive overview of the various septum deviations according to the nasal axis. Moreover, a treatment algorithm is suggested with technical details based on PR principles.

**Results:**

The directions and curvature of the cartilaginous deviation of crooked nose such as C-shaped, reverse C-shaped, straight axis deviations (I-shaped), and S-shaped are described. According to the deviation, a septoplasty classification (Type 1–Type 4) is suggested.

**Conclusions:**

On the basis of septal deviation, different PR techniques are proposed to achieve the desired straight nasal dorsum with an optimal functional outcome. Compared to the classical L-strut concept, the quadrangular cartilage remains preserved in the swinging door technique. The cartilage might be further used in the future for grafting in the hybrid structural/preservation technique if needed, ultimately saving rib cartilage and/or conchal cartilage. Finally, surgery time is reduced, and patient’s morbidity remains minimal.

**Level of Evidence III:**

This journal requires that authors assign a level of evidence to each article. For a full description of these Evidence-Based Medicine ratings, please refer to the Table of Contents or the online Instructions to Authors www.springer.com/00266.

## Introduction

A deviated nose is a common problem among patients for both cosmetic and functional reasons. Various factors such as trauma, congenital causes, and iatrogenic causes may have a role in the etiology of crooked nose deformity [1]. Differentiation must be done between bony trauma that exert extrinsic forces on nasal septum and direct cartilaginous trauma that cause cartilaginous fractures. The resulting deviation can be C-shaped, reverse C-shaped, linear I-shaped, or S-shaped [2]. One side of the dorsum in a C-shaped nose/reverse C-shaped nose is concave and the other is convex. The dorsum and tip in an I-shaped crooked nose (linear) are shifted to one side of the vertical midline of the face [3]. Depending on the nasal tip position, the patients can present with a S-shaped deformity [4]. Often, patients presenting with straight axis deviation (I-shape deviation) are also presenting with facial asymmetry, the nose being generally deviated to the shorter facial side. That being said, maxillary hypoplasia is often observed, requiring specific surgical procedures such as premaxillary grafts.

Unrecognized internal nasal septal deviations stand as a major reason for failed rhinoplasty outcomes [5]. Hence, precise analysis of a deviated nose is a crucial step in determining optimal surgical management. In most cases, the septal condition directs the deviation [3]. This term encompasses all clinical conditions involving deviation of the nasal pyramid from the midline of the face.

In the classic L-strut concept [6], a “component composite septorhinoplasty” is proposed to offer stable results. However, these procedures are usually done in an open approach and may require extracorporeal septoplasty with perpendicular plate transfer as graft supporting and straightening the anterior septum [4]. In addition, septal extension grafts may be required to support the tip, spreader grafts to stabilize the dorsum, asymmetric equalizing osteotomies, crisscross trans-osseous sutures to achieve a correct contour [7], and dorsal camouflage grafts. That being said, this approach with its associated procedures have their limitations and morbidities (“open roof syndrome”) [4, 8]. Thus, preservation rhinoplasty (PR) is suggested to be fast and potentially safer, without any loss of quality while achieving satisfactory results [4, 8]. Moreover, there is a paucity of data in the literature about septoplasty classifications and technical details in PR for various crooked noses.

The aim of this article is to provide a comprehensive overview of the various septum deviations with a suggested treatment algorithm by using PR principles. A septoplasty classification is suggested, which may help the clinicians in their day-to-day clinical practice.

## Materials and Methods

### Literature Search Strategy

A literature review was performed from 1982 to 2022 using the US National Institutes of Health’s PubMed database. The reference lists of all selected papers were further reviewed for potentially relevant analysis. Eligible reports were full papers written in

English language reporting on septal deviation. On the basis of septal deviation according to the nasal axis, we suggest a septoplasty classification Type 1–4 with various PR techniques (Table 1) [9–11] to achieve the desired straight nasal dorsum and optimal function.

**Table 1:** Septoplasty classification and technical details of deviated noses

Axial deviation	Septum deviation	Level of deviation	Surgical correction
C-shaped	Septum follows the dorsum on the same side with left-sided concavity	Left-sided concavity	Type 1: Modified swinging door endonasal technique: Disarticulation of the quadrangular cartilage, partial resection of the bony septum, repositioning of the quadrangular cartilage on the midline. Rasping on a convex bony cap [4] Type 2: Type 1 + septal subdorsal strip in more convex dorsum or in high straight deviated noses [4]
Reverse C-shaped	Septum follows the dorsum on the same side with right-sided concavity	Right-sided concavity	Type 1 Type 1: Swinging door technique [56] Type 2: Type 1 + septal subdorsal strip in more convex dorsum or in high straight deviated noses [4]
Linear (I-shaped)	Septum lies in opposite nasal fossa	The I-shaped deviation angle is measured as the angle between the vertical line drawn between the mid-point of the upper lip and the glabella mid-point and the line extending from the nasion to the nasal tip, representing the nasal dorsal axis[1]	Type 1: Swinging door technique [56] Asymmetric bony wedge resection (“Pisa Tower Concept”) [10, 13] Inferior removal of cartilaginous septal striprelease of the pyriform ligament and adjacent upper lateral/nasal junction on the short side of the nose [7] “Ballerina maneuver” [17] Excision of the sesamoid cartilages in the scroll area, no reattachment of the ligament on the shorter side [7]
S-shaped	Anteroposterior S-shape or cephalocaudal S-shape.[19]	Concave/convex deformity with bony pyramid deviations	Type 1: Swinging door technique [56] Type 2: Type 1 + septal subdorsal strip in more convex dorsum or in high straight deviated noses [4] Type 3 (Cottle’s technique): Mucoperichondrium and periosteum are undermined on both sides of the septum. Complete disarticulation of the cartilaginous septum from the bony septum is performed under video endoscopic vision. Anteroration of the quadrangular cartilage allows for dorsum lowering [4, 22, 23, 25, 26] Type 4: Complete radical excision and reinsertion after corrections in an extracorporeal way (endonasal total reconstruction, endonasal dorsum preservation rhinoplasty) [4]

## Results

### The C-Shaped Nose Deformity

**Fig. 1:**
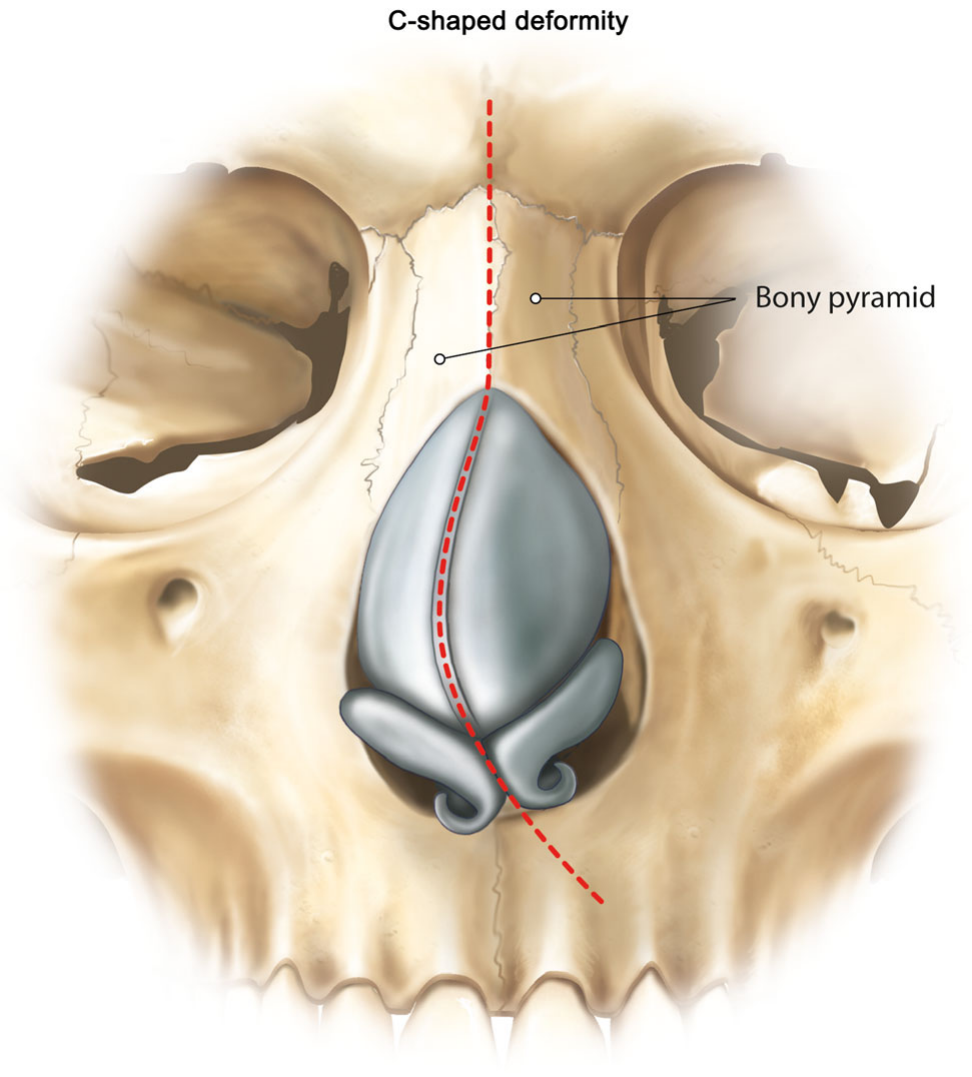
Illustration of the C-shaped nose deformity. The septum and bones are deviated in the opposite direction [4], the cartilaginous nasal dorsum has left-sided concavity in line with the dorsum [12]

The C-shaped crooked nose is defined by the presence of a single concavity (and convexity on the opposite side) [1]. The septum and bones are deviated in the opposite direction [4], the cartilaginous nasal dorsum has left-sided concavity in line with the dorsum (Fig. 1) [12]. The modified swinging door endonasal technique can be applied (Type 1) [4]. The quadrangular cartilage is disarticulated from the bony septum, followed by partial resection of the bony septum and sagittal repositioning of the quadrangular cartilage to the midline, affixing it to the spine with a secure suture through a drill hole [4, 7]. The mucoperichondrium is kept attached on the left side, acting as a central pillar and preserving vascularization [4]. If required, rasping can be performed on a convex bony cap [4]. No septum upper lateral cartilage (ULC) division is required. Lateral osteotomies are done after inner and outer subperiosteal elevation on the long side if required (“Pisa Tower Concept”) [13]. Direct percutaneous lateral osteotomies are performed on the short side, as well as transverse on both sides and radix osteotomies [4]. Some severe C-shaped deviations need extracorporeal septal correction and could be combined with spreader graft and osteotomies, if required (Table 1). However, very experienced surgeons can perform total septal extracoporeal reconstruction, even endonasally, together with dorsum PR [4, 14].

### The Reverse C-shaped Nose Deformity

**Fig. 2:**
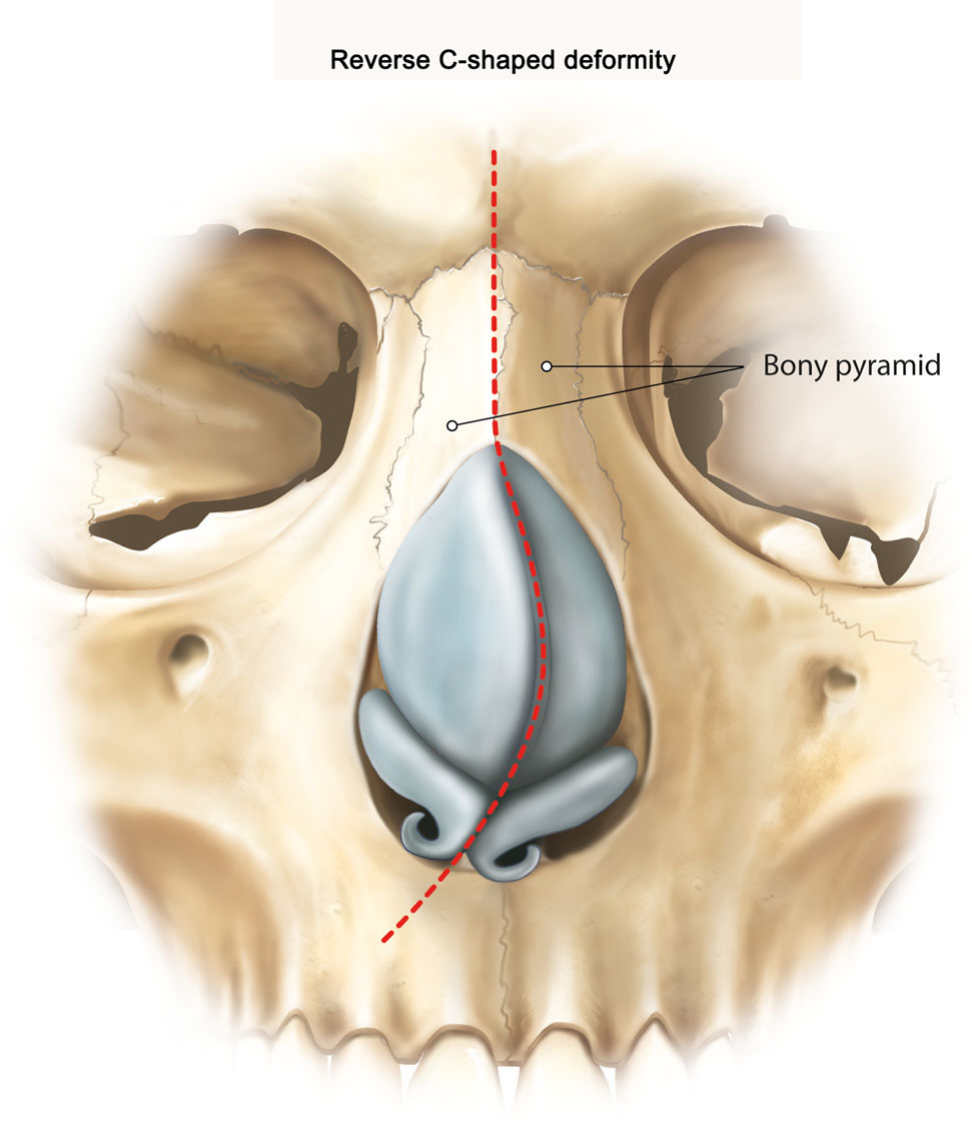
Illustration of the reverse C-shaped nose deformity. The septum and bones are deviated in the opposite direction [4], but the cartilaginous nasal dorsum has right-sided concavity [12]

As in the C-shaped nose deformity, the septum and bones are deviated in the opposite direction [4], but the cartilaginous nasal dorsum has right-sided concavity (Fig. 2) [12]. Karaca described the septal shift technique to correct crooked nose deformity, among also the reverse C-shaped nose deformity [15]. The modified swinging door technique can be applied as in the C-shaped nose deformity [4]. In cases of kyphotic noses or in high straight deviated noses, septal subdorsal strip resection can be performed (Type 2, Table 1) [4]. Otherwise, the principles of preservation by minimum cephalic resection, lateral crural underlay techniques, dome suturing, and lateral crural flare sutures are used to build a symmetric tip on a stable midline medial crural column [7].

### The I-Shaped Nose Deformity

**Fig. 3:**
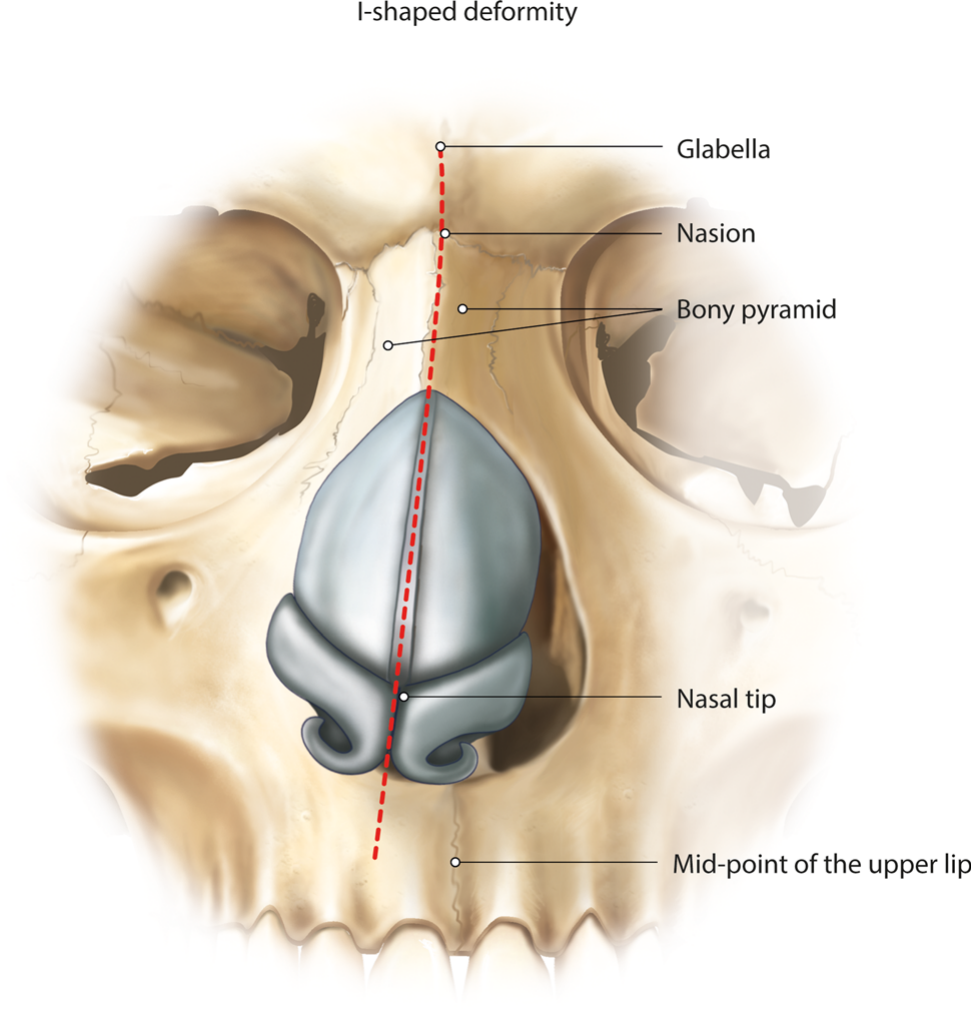
Illustration of the I-shaped nose deformity. The I-shaped crooked nose is the linear deviation of the septum to one side [1]. In this straight deviated noses (an ipsilateral deviation), the bony and cartilaginous deviations are on the same side [4]. The I-shaped deviation angle is measured as the angle between the vertical line drawn between the mid-point of the upper lip and the glabella mid-point and the line extending from the nasion to the nasal tip, representing the nasal dorsal axis [16]

The I-shaped crooked nose is the linear deviation of the septum to one side [1]. In this straight deviated noses (an ipsilateral deviation), the bony and cartilaginous deviations are on the same side (Fig. 3) [4]. The I-shaped deviation angle is measured as the angle between the vertical line drawn between the mid-point of the upper lip and the glabella mid-point and the line extending from the nasion to the nasal tip, representing the nasal dorsal axis [16]. By using the dorsal preservation principles, an asymmetric bony wedge resection with lowering of the bony pyramid onto the frontal process of the maxilla (the let-down osteotomy) can be performed (“Pisa Tower concept”) [10, 13]. A cartilaginous septal strip can be removed inferiorly to lower the dorsum together with the septum [4]. Release of the pyriform ligament and adjacent upper lateral/nasal junction on the short side of the nose should be performed, also described as the “Ballerina maneuver” [7, 17]. Excision of the sesamoid cartilages in the scroll area is recommended, and the ligament should not be reattached on the shorter side [7].

### The S-Shaped Nose Deformity

**Fig. 4:**
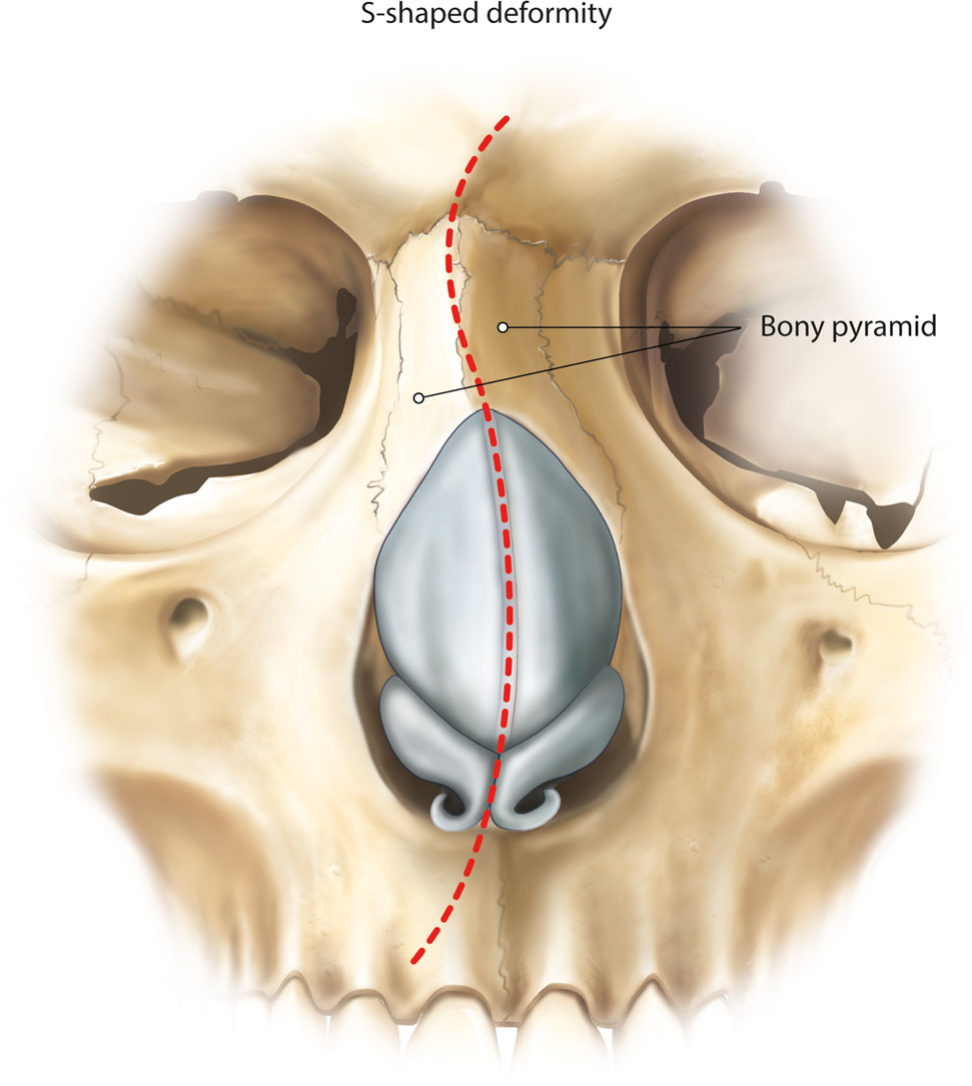
Illustration of the S-shaped nose deformity. S-shaped crooked nose is the repetition of this deformity more than once depending on the tip position [12]—causing multiple concave/convex areas with bony pyramid deviations [1]

S-shaped crooked nose is the repetition of this deformity more than once depending on the tip position [12]—causing multiple concave/convex areas (Fig. 4) [1]. The S-shaped nose deformity is the least common type within the septal deviation classification [18]. The deviation can be classified as anteroposterior S-shape or cephalocaudal S-shape [19]. Mobilization and straightening of the septum (caudal and dorsal aspect of the cartilaginous septum) can be technically difficult. Either endoscopic preservation septoplasty Type 1 or the Cottle’s technique can be performed (Type 3): [4, 8, 20, 21] Mucoperichondrium and periosteum are undermined on both sides of the septum. Then, complete disarticulation of the cartilaginous septum from the bony septum is performed under video endoscopic vision. Anteroration of the quadrangular cartilage allows for dorsum lowering [4]. Many variations following Cottle’s technique have been described that are reliable procedures, such as the SPAR (= Septum Pyramidal Adjustment and Repositioning) technique by Dewes [22] and the Tetris procedure by Neves [23]. Most [24] introduced an intermediate flap, whereas Neves described a squared high septal flap, allowing for stable dorsum lowering and fixation [23]. Finocchi repopularises the original Cottle’s technique called “SPQR” (= Simplified Preservation of Quick Rhinoplasty) [25]. Kovacevic designed a septal triangular Z-plasty section that he named “subdorsal Cottle” [26]. The maxillary crest is resected using a fine bone rongeur. In cases where the septum distortions do not allow any conservation nor direct repositioning, a complete radical excision and reinsertion after correction in an extracorporeal way is suggested (endonasal total reconstruction, endonasal dorsum PR, and Type 4 septoplasty) [4, 27–31]. Even in these difficult cases, it is possible to preserve the vaults [4, 8]. Keeping intact the quadrangular cartilage allows either for further subdorsal septal strip resection if a hump reduction is planned, or for only direct reposition on the midline if no profile lowering is desired. In severe septal deformities, the perpendicular plate of ethmoid can be harvested and used to stabilize the anterior septum, with a fixation on the anterior nasal spine [4, 27, 32]. After septal correction, other procedures might be needed to straighten the crooked nose (medial and/or lateral osteotomies, full or hinge radix osteotomies, and splints) (Table 1). An oblique radix cut may be used to create a rotational hinge of the dorsum, therefore preventing posteriorly displacement of the radix point [7].

## Discussion

The deviated nose represents a complex cosmetic and functional problem [33]. Thus, correction of the crooked nose with long-term functional and aesthetic success remains a major challenge for the rhinoplasty surgeon [1]. Crooked nose deformity can be defined as the deviation of the nasal bone and cartilage pyramid relative to the facial sagittal plane [34]. Therefore, treatment of the deviated nose should involve the recognition and correction of all underlying pathologies, and careful preoperative analysis and planning are mandatory. For the permanent correction of a crooked nose, it is necessary to release all the relevant intrinsic and extrinsic pathological subunits and to relocate them appropriately [35].

A simple and descriptive classification of the deviated nose would be of great benefit to the analysis and characterization of the pathologic abnormalities. Clearly, there is a paucity of data in the literature about its classification and treatment, especially using PR principles. Four types of crooked nose deformity are defined based on the direction of curvature of the deviation: A C-shaped or reverse C-shaped deviation, which are concave and convex deviations of the nasal pyramid; an I-shaped deviation, which is a linear deviation of the nasal pyramid; and an S-shaped deviation, in which concavity and convexity are on the same side of the nasal pyramid [2]. Ellis and Gilbert [36] used three categories of nasal deviation: (1) At the nasion, (2) at the level of the rhinion, and (3) combinations of the two. Rohrich et al. [18] also suggested three basic types of nasal deviation: (1) Caudal septal deviations, (2) concave dorsal deformities, and (3) concave/convex dorsal deformities. The most common type of dorsal deviation in their study was their type 2, of which there were 2 subtypes: C-shaped deformity, with left-sided concavity, and reverse C-shaped deformity, with right-sided concavity. The least common type in their classification was the S-shaped dorsal deformity with bony pyramid deviations with concave/convex deformity [18]. According to Neves’ analysis, three main dorsal segments are critical [23]. These include three options for the radix (normal, high, and low), three options for the keystone area (straight, convex, kyphotic including Lazovic [37] S-shaped nasal bones), and two options for the supratip segment (straight or curved), producing a complex of 18 variations in dorsum profile lines (DPL) [11].

Congenital anomalies, trauma (especially during childhood), and iatrogenic causes may play a role in the etiology of crooked nose. A partial or complete destruction and absorption of the nasal bones may occur after childhood injuries and infections, and this may be accompanied by normal or accelerated growth of the septum and its associated structures [8].

Surgical management of the deviated nose involves septal correction and bony pyramid manipulation after osteotomies. A cone beam CT scan should be performed routinely preoperatively to allow 3-D examination, nasal valve analysis, and facial reconstruction layer by layer [4]. Moreover, looking at the septoplasty procedure’s safety, one can measure the distance between the rhinion and the skull base, the lamina cribriformis aspect, asymmetry and partial dehiscence [4].

The principles of PR are to respect, conserve or restore the soft tissue envelope ligaments, to minimize the resection of cartilage through reorientation and to keep the dorsal continuity of the patient’s own bridge, hence minimizing the “open roof syndrome” [7, 8]. Although initially described as an endonasal procedure, PR can be performed via open or closed approaches [7]. With the widespread adoption of PR techniques, surgeons began to realize the importance of dorsal hump configuration and the aesthetic and functional consequences of modifying the K-area [10, 38]. Hence, PR is a more conservative and much less aggressive approach. The possibility of obtaining a totally smooth nasal contour after the correction of a hump, leaving this area intact, was championed by Saban [4, 10, 11] and Cakir [39] and has garnered the enthusiasm of many surgeons around the world. This goes in line with recent publications, where it has been shown that a high patient satisfaction can be achieved after PR as evaluated by the Rhinoplasty Outcomes Evaluation Questionnaire (= ROE) [40–43]. Nevertheless, hybridization between preservation and structural rhinoplasty can be performed including septal extracorporeal reconstruction or the swinging door technique together with dorsum PR (“hybrid operation”) [4, 7].

In practice, surgical procedures are performed “from depth to surface” to avoid the bleeding related to the rhinoplasty which makes video endoscopic surgery difficult. The recommended surgical sequence is: endonasal endoscopic procedures; “preservation” septoplasty; asymmetric dorsum–“lateral push over”; tip surgery, if required, and ancillary procedures [4]. Deviated noses may also involve the turbinates, which should be addressed accordingly [4]. Although it may be possible to preserve many of the ligaments in deviated noses, some may have to be modified or even released. In particular, the position of insertion of the vertical scroll ligament on the short side of the deviated nose will be different with the repositioned nasal pyramid [7]. Sharp dissection of the upper lateral, nasal bone overlap parallel to the upper lateral cartilage and extending up to within 5 mm of the dorsum will allow the vertically short side to elongate, hereby minimizing the risk of redeviation (“Ballerina maneuver”) [7, 17]. Moreover, it has been suggested to excise the sesamoid cartilages in the scroll area and not to reattach the ligament particularly on the shorter side [7]. This is due to the fact that discrepancy in the skin envelope may recreate a deformity and consequently, the scroll cartilages may displace cranially, producing an unfavorable supratip bulge [7]. Hereby, a rotational lengthening of the short middle third is achieved without requiring to reattach the scroll area on the longer side. Lastly, there may be pre-existing nostril asymmetry and a need for differential alar base reduction. In principle, the more vertical side of the nasal tip will need to be lengthened to allow the dome to be approximated in the midline with its opposite side [7]. Release of the nasal tip from the muscles around the piriform aperture may be also required together with augmentation of the premaxilla under the alar using free segments of cartilage or diced cartilage injected via an incision in the floor of the nasal vestibule in a similar fashion to augmenting a depressed alar sidewall in a cleft nose [7].

An important element toward correction of deviated nose is the caudal septoplasty, which involves a wide dissection and mobilization of the caudal septum to the midline in a “swinging door” fashion [44, 45], with excision of the overhanging part. General principles include preservation of the septal cartilage integrity where unnecessary cartilage resection is avoided and vitality by keeping intact the mucoperichondrium attachments on one side [4]. The osseocartilaginous junction should be freed to allow sagittal repositioning of the quadrangular cartilage on midline. Ultimately, the endoscope should be used for precise dissection in depth [4].

The described techniques call for creation of a wide pocket in the area of the anterior nasal spine and maxillary crest to allow the caudal septum to move toward the midline. Pastorek described his “modified swinging door” technique in which the caudal septum is flipped over the nasal spine, which acts as a doorstop holding the septum in the midline. Hereby, the main goal is to preserve as much as possible the whole quadrangular cartilage by just repositioning it on the midline onto the maxillary crest. Only partial resections are performed to allow for adjusting its height to the nasal height [4]. A suture is then used to secure the septum to the nasal spine [46].

To date, nose deviation seemed to be an exclusion criterion for the dorsal preservation technique. A septoplasty with an L-strut (L-strut concept [6]) is usually performed before a nasal osteotomy, but spreader graft, septal extension graft and suture in-placement should be done after the osteotomy for dorsal axis correction with a proper contour and tip position [47]. This method fundamentally serves to strengthen the middle nasal vault, and hence prevents postoperative collapse. It also proves immediately functionally better by broadening the angle of the internal nasal valve, thus increasing respiratory airflow [41–43, 47, 48]. However, the “Pisa Tower concept” [13] helps to understand that septoplasty procedures can be adapted to the requirements for the bony and cartilaginous surgical procedures according to their difficulties, leading to a straight nose with a straight septum and sufficient ventilation. Eventually, the swinging door septoplasty preserves the quadrangular cartilage. It might be further used in the future for grafting in the hybrid structural/preservation technique if needed, ultimately saving rib cartilage and/or conchal cartilage. Finally, surgery time is reduced, and patient’s morbidity remains minimal.

It has been reported that the combination of asymmetric spreader grafts with spreader flaps might be successful in correction of the nasal deviation in C-shaped crooked nose surgery, but most significantly in I-shaped crooked nose surgery [49]. *In situ* septal corrections (ISSCs) are extremely effective approaches for the correction of the nasal septum and the external nose in patients with a deviated nose [1]. However, because of cartilage memory, these procedures may not always be sufficient for correction of severely deviated noses in particular. Therefore, extracorporeal septoplasty (ECS) has been suggested in rhinoplasty as a more basic correction method for severely deviated septum [1, 27, 28, 32]. However, some authors have claimed that this technique has a risk of long-term destabilization in the keystone area and may lead to aesthetic problems such as saddling in the nasal dorsum or irregularities [50]. Hence, in combination with swinging door septoplasty, the ”Pisa Tower Concept” is able to correct a difficult crooked nose without the need for an extracorporeal septoplasty [4, 10, 13].

Recently, surgeons have begun using piezoelectric-powered ultrasonic instruments (PEIs) for the management of the bony vault and lateral osteotomies [51, 52]. PR can be performed with piezoelectric instrumentation and different insets. PEIs act selectively on the bone and the fracture lines created by PEIs are very accurate and eliminate the risk of radiating fracture lines encountered with traditional instrumentation [53]. A big advantage of piezo-assisted septoplasty according to Gerbault et.al. [53] is the safe correction of high septal deviations. A small strip of perpendicular plate can be removed without twisting the septum. Also, there is a much less risk of a radiated fracture to the skull base. Once the bony excess of the septum is removed, the remaining part can be medialized [53].

When we correct the deviated nose, the general surgical principles suggested by previous rhinoplasty surgeons are followed accordingly [18, 54]. Nevertheless, a failure rate 4.76% [13] up to 11% [3] has been reported in the literature. Possible reasons for these failures may include improper preoperative evaluation, failure to understand and compensate for the dynamics of the cartilage, and faulty surgical execution [55]. Half of the failures were attributable to the conservatism of an endonasal approach. Thus, for the proper management of the deviated nose, Jang et al. [3] recommend a more aggressive approach through an open rhinoplasty incision, which provides for a better intraoperative diagnosis and more precise execution of the various maneuvers required to correct the deviated nose [3]. That being said, hybridization between preservation and structural rhinoplasty can be performed by an experienced PR surgeon (“hybrid operation”) [7].

Our article has limitations: There is a lack of objective measurements such as rhinomanometry and validated subjective patient reported outcome measures. Nevertheless, it has just recently been reported that a high patient satisfaction after PR can be achieved, as evaluated by the ROE, which goes in line with aesthetics and function [41–43]. Lastly, further studies are needed by using also the septal extracorporeal reconstruction technique, even endonasally together with dorsum PR [4].

## Conclusion

The nasal septum deviation condition in crooked noses is a very widespread pathology, which often leads to nasal airway obstruction. We provide a septoplasty classification with technical details of the various septum deformities according to the nasal axis. By applying a treatment algorithm respecting the PR principles, a satisfactory outcome can be achieved with low morbidity. Compared to the L-strut concept, the osseocartilaginous vault and quadrangular septum remain largely preserved in the swinging door technique.
